# Comparison of the Carcinogenic Effect of X-Irradiation with Radioactive Iodine on the Rat's Thyroid

**DOI:** 10.1038/bjc.1957.10

**Published:** 1957-03

**Authors:** I. Doniach

## Abstract

**Images:**


### COMPARISON OF THE CARCINOGENIC EFFECT OF X-IRRADIATION WITH RADIOACTIVE IODINE ON THE RAT'S THYROID

I. DONIACH

*From the Pathology Department, the Postgraduate Medical School of London, DuCane Road, W.12*

Received for publication December 15, 1956

RATS injected with 30 $\mu$C radioactive iodine ($I^{131}$) and kept on methylthiouracil for the succeeding 15 months show adenomatous replacement of the thyroid gland and occasional thyroid carcinomas ; unirradiated controls treated with methylthiouracil for 15 months develop a moderate number of adenomas but no carcinomas (Doniach, 1950 and 1953). The radiation dose, which is comparable to that used in the treatment of Graves' disease, varies extremely widely within the rat's thyroid gland. The following experiment is an attempt to find out whether radiation in the lower part of the dosage range, given before the goitrogenic treatment, is effective or not in initiating carcinogenesis. Dosage uniformity within the thyroid was obtained by irradiation with X rays. Comparison was made between the carcinogenic effect of 30 $\mu$C $I^{131}$ with a single exposure of the thyroid to 1100 rads X rays. This dose of X rays was chosen because we had found previously in a biological assay that it was comparable with 30 $\mu$C $I^{131}$ in its potency to inhibit the response of the rat's thyroid to a goitrogenic challenge given 4 months after the irradiation (Doniach and Logothetopoulos, 1955 ; Abbatt, Doniach, Flanders and Logothetopoulos, 1957).

### MATERIAL AND METHODS

One hundred and sixty animals were used. They were black and white hooded male and female rats from a closed colony of the hooded Lister strain, fed on "Research" rat cubes with additional bread and greens. The 4-methyl-2-thiouracil (B.D.H.) was given as a saturated solution in the drinking tap water, made up once weekly by suspending 1 g. of the compound in each litre of water. The radioactive iodine, $I^{131}$, was injected intraperitoneally, carrier free as iodide in 1 ml. water. For X-irradiation deep anaesthesia was temporarily induced with ether vapour followed by intramuscular injection of 2·5 mg. Largactil (Chlorpromazine). The anaesthetized animals were immediately confined in a special holder and irradiated as described by Abbatt *et al.* (1957), the site of the thyroid having been marked with ink on the skin of the front of the neck. The X ray beam was defined by a perforated lead shield to a diameter of 1·3 cm. applied to the central surface of the neck immediately over the thyroid and portion of trachea between the 2 lobes. The physical factors were ; 190 kV. X rays, 6 m.a., filtered by $\frac{1}{2}$ mm. Cu. and 1 mm. Al. The dose rate, taking the centre of the thyroid to be 8 mm. below the skin surface, was 150 rads/min. measured by means of a BD2 type condenser ionization chamber with the small air voume placed at this depth. 1100 rads were delivered to the thyroid of each rat. The irradiation was carried out by P. Howard Flanders of the Experimental Radiopathology Research Unit of the Medical Research Council at Hammersmith Hospital. The animals averaged 3 months of age at the beginning of the experiment and were killed by coal gas 15 months later. The trachea and attached thyroid were fixed in Helly's fluid; the thyroid was then dissected off and weighed. When the thyroid was pathologically adherent to neighbouring structures, the actual adhesions to cervical connective-tissue, muscle or trachea were dissected off attached to the thyroid gland. Each thyroid was bisected in a horizontal plane through the isthmus and the two halves embedded in a single block. After trimming, a ribbon of about 12 serial sections was cut at 5 $\mu$ and mounted on 3 slides. One slide from each block was stained by haemalum and eosin, the spares were used for extra stains.

The rats were divided into 6 groups as follows: (1) Controls. (2) 30 $\mu$C $I^{131}$. (3) 1100 rads X rays. (4) Methylthiouracil in the drinking water for the duration of the experiment. (5) 30 $\mu$C $I^{131}$ followed after 3 days by methylthiouracil until the end of the experiment. (6) 1100 rads X rays followed after 24 hours by methylthiouracil till the end of the experiment. The animals on methylthiouracil were given a rest from this toxic drug for 8 weeks during the 9th and 10th month.

Six months after the start of the experiment 3 males and 2 females taken at random from each of groups (1), (2) and (3) were put on to propylthiouracil in the drinking water, 6 mg./10 ml., and killed at the end of 12 days. Their thyroids were removed and weighed in order to confirm an equivalent inhibition of induced goitrogenesis in the $I^{131}$ and X-irradiated animals.

### RESULTS

The subsidiary test described above removed 15 rats from the original 160. A further 33 animals died and were discarded. Final histology was carried out on 112 rats, 66 males and 46 females.

### *Inhibition of goitrogenesis*

The results, given in Table I, show a definite and more or less equivalent reduction in goitrogenic response in all the irradiated rats. The average weight of the control goitres was 46 mg., that of the $I^{131}$ group 27 mg. and of the X-irradiated group 29 mg.

### *Main experiment*

The findings are summarized in Table II–VII. The histological findings were the same as those described previously (Doniach, 1950 and 1953). No morphological

---

EXPLANATION OF PLATE

FIG. 1.—Thyroid of female X ray rat 9B showing carcinomatous infiltration of the capsule. × 60.

FIG. 2.—Thyroid of female $I^{131}$ methylthiouracil rat 16C showing adenomatous replacement and carcinomatous permeation of a capsular vein. × 60.

FIG. 3.—Thyroid of male X ray methylthiouracil rat 5C showing adenomatous replacement and carcinomatous permeation of a capsular vein. × 60.

FIG. 4.—Perithyroid tissue of female X ray methylthiouracil rat 6B showing extension of adenomatous growth into cervical muscle and connective tissue. × 20.

FIG. 5.—Cervical muscle of female X ray methylthiouracil rat 6C showing permeation of an extrathyroidal vein with a thyroid carcinomatous acinus. × 120.

FIG. 6.—Thyroid of female X ray methylthiouracil rat 7A showing carcinomatous permeation of extracapsular veins. × 120.

1 2 3 4 5 6

Doniach.

differences were found between the effects of $I^{131}$ administration and X-irradiation. Adenomas indicate discrete nodules of thyroid tissue clearly separate and of a different morphology from surrounding parenchyma. Nodules smaller than 0·5 mm. in diameter and discrete foci of thyroid tissue which differed only slightly from surrounding parenchyma were regarded as border line tumours and were not included in the final assessment. Adenomatous replacement of one or both lobes, designated ++++ in the tables, refers to the presence of large and numerous adenomas associated with distortion and diminution of surrounding parenchyma and usually with gross fibrous thickening of the thyroid capsule. Carcinoma was diagnosed in all but one instance by the finding of tumour tissue lying within the lumen of capsular or extra-capsular veins. The exceptional carcinoma showed massive invasion of the capsule by dedifferentiated tumour cells. The findings are not quantitatively comparable with those of the author's previous similar experiments because this time the thyroids were not serially sectioned (Doniach, 1950) or cut at 6 levels (Doniach, 1953). This has reduced the number of adenomas counted but has not materially altered the quantitative findings in thyroids showing adenomatous replacement or malignant change.

There were 17 rats in the controls (Table II). Three of them showed small

TABLE I.—*Goitrogenic Response 6 months after Irradiation*

| | Controls | | 30 μC $I^{131}$ | | 1100 rad X rays | |
|---|---|---|---|---|---|---|
| Sex | Body weight (g.) | Thyroid weight (mg.) | Body weight (g.) | Thyroid weight (mg.) | Body weight (g.) | Thyroid weight (mg.) |
| M. | 300 | 55 | 330 | 31 | 300 | 30 |
| ,, | 340 | 44 | 360 | 32 | 320 | 28 |
| ,, | 300 | 42 | 280 | 22 | 260 | 31 |
| F. | 240 | 48 | 230 | 24 | 210 | 25 |
| ,, | 200 | 41 | 250 | 25 | 210 | 30 |

All rats were killed at the end of a 12-day course of propylthiouracil 6 mg./10 ml. in the drinking water.

TABLE II.—*Controls*

| Rat | Sex | Body weight (g.) | Thyroid weight (mg.) | Micro-adenomas |
|---|---|---|---|---|
| 23A | F. | 220 | 23·0 | + |
| 23B | ,, | 215 | 13·0 | — |
| 23C | ,, | 195 | 25·1 | — |
| 23D | ,, | 230 | 28·1 | — |
| 23E | ,, | 205 | 20·1 | — |
| 24A | ,, | 225 | 18·0 | + |
| 24B | ,, | 225 | 22·6 | — |
| 24C | ,, | 235 | 29·8 | — |
| 25A | M. | 295 | 24·6 | — |
| 25B | ,, | 295 | 28·4 | — |
| 25C | ,, | 335 | 33·5 | — |
| 25D | ,, | 350 | 33·7 | + |
| 25E | ,, | 345 | 32·9 | — |
| 26A | ,, | 325 | 22·5 | — |
| 26B | ,, | 325 | 32·4 | — |
| 26C | ,, | 335 | 27·9 | — |
| 26D | ,, | 290 | 25·9 | — |

Rats with the same number in the first column are from the same cage.

+ Represents the finding of scattered microadenomas.

collections of " solid " cellular follicles with occasional microfollicle development. There were no adenomas comparable with those found in the other groups.

Adenomas were present in 4 of the 22 rats of the $I^{131}$ group (Table III) and in 4 of the 13 rats in the X-irradiated group (Table IV). One of the thyroids in the latter group weighed 160 mg. It showed adenomatous replacement of the left lobe with carcinomatous invasion of the capsule (Fig. 1). This thyroid was 5 to 10 times heavier than the other glands in both of these groups.

Of the 14 rats in the methylthiouracil group (Table V) 10 animals showed adenomas, the largest measuring 2·0 mm. across. There was no adenomatous replacement of any lobe.

TABLE III.—30 *μC* $I^{131}$

| Rat | Sex | Body weight (g.) | Thyroid weight (mg.) | Number of adenomas | Diameter of largest adenoma (mm.) |
|---|---|---|---|---|---|
| 10A | M. | 305 | 14·5 | 1 | 0·5 |
| 10B | ,, | 350 | 27·8 | 1 | 0·5 |
| 10C | ,, | 350 | 19·9 | — | — |
| 10D | ,, | 390 | 20·7 | — | — |
| 11A | ,, | 405 | 19·0 | 1 | 0·5 |
| 11B | ,, | 270 | 17·5 | — | — |
| 11C | ,, | 370 | 37·8 | 2 | 1·5 |
| 11D | ,, | 280 | 20·3 | — | — |
| 12A | ,, | 240 | 14·0 | — | — |
| 12B | ,, | 345 | 22·6 | — | — |
| 12C | ,, | 345 | 21·7 | — | — |
| 12D | ,, | 355 | 20·3 | — | — |
| 13A | F. | 230 | 20·3 | — | — |
| 13B | ,, | 265 | 16·2 | — | — |
| 13C | ,, | 270 | 18·3 | — | — |
| 13D | ,, | 190 | 13·7 | — | — |
| 17A | M. | 385 | 25·0 | — | — |
| 17B | ,, | 375 | 26·2 | — | — |
| 19A | F. | 220 | 15·8 | — | — |
| 19B | ,, | 215 | 16·8 | — | — |
| 19C | ,, | 260 | 17·5 | — | — |
| 19D | ,, | 235 | 20·9 | — | — |

TABLE IV.—1100 *rads X rays*

| Rat | Sex | Body weight (g.) | Thyroid weight (mg.) | Number of adenomas | Diameter of largest adenoma (mm.) |
|---|---|---|---|---|---|
| 1A | M. | 290 | 21·5 | 1 | 0·5 |
| 1B | ,, | 370 | 33·4 | — | — |
| 2A | ,, | 335 | 29·0 | — | — |
| 3A | F. | 210 | 14·5 | — | — |
| 3B | ,, | 245 | 21·1 | — | — |
| 3C | ,, | 230 | 26·3 | — | — |
| 3D | ,, | 265 | 22·9 | — | — |
| 4A | M. | 345 | 26·2 | 1 | 1·0 |
| 4B | ,, | 325 | 17·3 | — | — |
| 4C | ,, | 360 | 28·1 | 2 | 0·5 |
| 9A | F. | 255 | 15·3 | — | — |
| 9B | ,, | 245 | 160·0 | ++++ Ca. | 4·5 |
| 9C | ,, | 250 | 28·8 | — | — |

++++ represents adenomatous replacement of one or both lobes.

Ca. represents malignancy.

There were 24 rats in the group given 30 $\mu$C $I^{131}$ followed by methylthiouracil (Table VI). All except 1 showed adenomas, 16 showed adenomatous replacement of the thyroid and malignancy was diagnosed in 5 (Fig. 2). The heaviest thyroid was 385 mg. and the largest adenoma 5·0 mm. in diameter. All except 1 of the 22 rats given 1100 rads X-rays followed by methylthiouracil (Table VII)) showed adenomas. There was adenomatous replacement of the thyroid in 17 animals and evidence of maligancy in 7 (Fig. 3, 4, 5, 6). The heaviest gland weighed 566 mg. (Fig. 4) and the diameter of the largest adenoma was 5·0 mm.

TABLE V.—*Methylthiouracil*

| Rat | Sex | Body weight (g.) | Thyroid weight (mg.) | Number of adenomas | Diameter of largest adenoma (mm.) |
|---|---|---|---|---|---|
| 20A | F. | 215 | 114 | 3 | 0·75 |
| 20B | ,, | 135 | 50 | — | — |
| 20C | ,, | 180 | 89 | 1 | 0·5 |
| 20D | ,, | 255 | 266 | 2 | 1·0 |
| 20E | ,, | 205 | 207 | 3 | 0·75 |
| 21A | M. | 220 | 161 | — | — |
| 21B | ,, | 240 | 245 | 2 | 2·0 |
| 21C | ,, | 230 | 272 | 7 | 1·0 |
| 21D | ,, | 210 | 165 | 2 | 0·75 |
| 22A | ,, | 240 | 204 | — | — |
| 22B | ,, | 215 | 102 | — | — |
| 22C | ,, | 295 | 208 | 2 | 1·0 |
| 22D | ,, | 305 | 298 | 4 | 1·5 |
| 22E | ,, | 315 | 211 | 2 | 0·75 |

TABLE VI.—*30 $\mu$C $I^{131}$ and Methylthiouracil*

| Rat | Sex | Body weight (g.) | Thyroid weight (mg.) | Number of adenomas | Diameter of largest adenoma (mm.) |
|---|---|---|---|---|---|
| 14A | M. | 215 | 165 | 4 | 2·0 |
| 14B | ,, | 205 | 91 | 12 | 1·0 |
| 14C | ,, | 245 | 69 | ++++ | 1·5 |
| 14D | ,, | 245 | 127 | ++++ | 3·5 |
| 15A | F. | 215 | 180 | ++++ Ca. | 2·0 |
| 15B | ,, | 230 | 96 | ++++ | 2·5 |
| 15C | ,, | 200 | 116 | ++++ Ca. | 2·5 |
| 16A | ,, | 165 | 35 | 4 | 1·0 |
| 16B | ,, | 145 | 38 | 3 | 0·5 |
| 16C | ,, | 200 | 229 | ++++ Ca. | 3·5 |
| 16D | ,, | 205 | 75 | ++++ | 1·5 |
| 18A | M. | 270 | 141 | ++++ | 2·0 |
| 18B | ,, | 235 | 360 | ++++ | 3·5 |
| 18C | ,, | 245 | 245 | ++++ Ca. | 4·0 |
| 18D | ,, | 255 | 139 | ++++ | 2·5 |
| 31A | ,, | 260 | 212 | ++++ | 4·0 |
| 31B | ,, | 300 | 385 | ++++ | 5·0 |
| 32A | F. | 215 | 39 | — | — |
| 32B | ,, | 200 | 328 | ++++ | 4·5 |
| 32C | ,, | 205 | 165 | ++++ Ca. | 3·5 |
| 33A | M. | 295 | 207 | ++++ | 2·0 |
| 33B | ,, | 265 | 52 | 1 | 0·5 |
| 33C | ,, | 260 | 143 | 4 | 1·0 |
| 33D | ,, | 255 | 167 | 8 | 1·5 |

++++ and Ca. as in Table IV.

TABLE VII.—1100 *rads X rays and Methylthiouracil*

| Rat | Sex | Body weight (g.) | Thyroid weight (mg.) | Number of adenomas | Diameter of largest adenoma (mm.) |
|---|---|---|---|---|---|
| 5A | M. | 245 | 67 | ++++ | 2·5 |
| 5B | ,, | 285 | 98 | 2 | 1·0 |
| 5C | ,, | 180 | 148 | ++++ Ca. | 3·0 |
| 6A | F. | 220 | 193 | ++++ | 3·0 |
| 6B | ,, | 200 | 566 | ++++ Ca. | 4·0 |
| 6C | ,, | 195 | 193 | ++++ Ca. | 4·5 |
| 7A | ,, | 200 | 276 | ++++ Ca. | 3·0 |
| 7B | ,, | 185 | 132 | ++++ | 2·5 |
| 7C | ,, | 220 | 186 | ++++ | 2·0 |
| 8A | M. | 230 | 238 | ++++ | 3·5 |
| 8B | ,, | 235 | 93 | ++++ | 2·5 |
| 8C | ,, | 210 | 236 | ++++ | 2·5 |
| 27A | ,, | 305 | 153 | ++++ | 3·5 |
| 27B | ,, | 255 | 72 | 1 | 1·0 |
| 27C | ,, | 255 | 172 | ++++ Ca. | 4·0 |
| 27D | ,, | 275 | 115 | ++++ | 2·5 |
| 28A | ,, | 215 | 375 | ++++ Ca. | 5·0 |
| 28B | ,, | 185 | 149 | ++++ | 3·5 |
| 28C | ,, | 230 | 53 | 5 | 1·5 |
| 28D | ,, | 255 | 35 | 3 | 0·5 |
| 29A | F. | 175 | 138 | ++++ Ca. | 3·0 |
| 29B | ,, | 320 | 121 | — | — |

++++ and Ca. as in Tables IV and VI.

### DISCUSSION

The findings show a close parallel between the effect of 1100 rads X rays and 30 $\mu$C $I^{131}$ in adenoma production in the thyroid and carcinoma production when followed by the administration of methylthiouracil for 15 months.

Due to variations between uptake and retention of iodine and distance from the gland periphery of thyroid follicles it is ony possible to express the dosage of radiation to the rat thyroid from administered radioactive iodine as a range. In our animals injected intraperitoneally with 30 $\mu$C $I^{131}$ we regarded the range to lie between 2000 and 24,000 rads (Abbatt *et al.*, 1957). I thought (Doniach, 1953) that the carcinogenic activity of 30 $\mu$C $I^{131}$ might have been initiated by *beta* radiation of the order of 5000 to 10,000 rads and promoted by the subsequent pituitary thyrotrophic hormone (T.S.H.) stimulation induced by prolonged thiouracil. The dosage of 5000 to 10,000 rads from *beta* rays has been shown to be carcinogenic to the skin of experimental animals (Raper, Henshaw and Snider, 1951 ; Glücksmann, 1951). When we found that 1100 rads X rays and 30 $\mu$C $I^{131}$ were equivalent in their potency to inhibit hyperplasia of the thyroid (Doniach and Logothetopoulos, 1955) I considered that this dose of X-irradiation which lies well below 5000 rads might be non- or only very weakly carcinogenic. If so, X rays might be used for the treatment of Graves' disease with little risk of future carcinoma development. However, the results have proved otherwise and require further analysis.

It is now generally accepted that prolonged stimulation with T.S.H. alone leads eventually to carcinoma formation in the rat's thyroid (Bielschowsky, 1955 ; Axelrad and Leblond, 1955). The excess T.S.H. output may be produced by administrationof goitrogens (Purves and Griesbach, 1947) or by iodine deficiency (Axelrad and Leblond, 1955).

*Carcinogenic action of T.S.H.*

The development of tumours as a result of prolonged excessive stimulation of the thyroid by the pituitary is an example of experimental carcinogenesis without the agency of chemical or physical carcinogens in the usual sense. Though T.S.H. is a physiological secretion and its excessive output in gross iodine deficiency is a physiological compensatory phenomenon the resultant state of the thyroid is remarkably different from the normal resting gland. In the usual laboratory environment most of the cells of the adult rat thyroid last the animal's lifetime without renewal (Leblond and Walker, 1956). Goitrogen treatment leads to a tremendous wave of mitoses and a maintained marked cellular hypertrophy. With the passage of time multicentric adenomas appear in increasing numbers, grow and eventually show signs of malignancy. In T.S.H. carcinogenesis, therefore, it appears that an abnormally high rate of fission induced in normal cells has led to neoplasia. Analagous examples of tumour development are seen in ovarian auto-implants into the spleen of spayed mice, testicular auto-implants into the spleen of castrated rats, mammary glands of oestrogen-treated susceptible mice and possibly in the regenerating livers of cirrhotics.

*Carcinogenic action of irradiation and summation with thyroid hyperplasia*

Morphological evidence has been found in the thyroid (Maloof, Dobyns and Vickery, 1952; Doniach and Logothetopoulos, 1955) and pituitary (Goldberg and Chaikoff, 1950; Doniach, 1953) that there is a maintained increased output of T.S.H. following the administration of $I^{131}$. This appears to be a compensatory phenomenon which enables the damaged thyroid to put out a normal quantity of thyroid hormone at the expense of diminished hormone storage. Thus, the adenoma formation observed in the present experiment in the $I^{131}$ and X-irradiated rat groups (2) and (3), may have resulted partly from the post irradiation increased output of T.S.H. However, after 30 $\mu$C $I^{131}$ the average thyroid follicle cell height rises by only 16 per cent (Doniach and Logothetopoulos, 1955) whereas after methylthiouracil the cells are more than doubled in height. Since the increase in cell height is an index of the level of T.S.H. secretion, it follows that the T.S.H. level is far higher following methylthiouracil than after 30 $\mu$C $I^{131}$. It is possible that the increased numbers of adenomas in the methylthiouracil-treated rats, group (4), as compared with the irradiated ones, groups (2) and (3), reflects the greater output of T.S.H. On the other hand the increase in T.S.H. output in the irradiated animals appears to be comparatively slight and therefore unlikely to account by itself for adenoma production within 15 months. The tumours more probably result from the effects of summation of radiation with increased T.S.H. This is supported by the finding of one carcinoma in the X-irradiated group (3) in contrast to the absence of carcinomas in the methylthiouracil rats group (4), killed after 15 months, in this and in previous experiments (Doniach, 1950 and 1953) in spite of the considerably higher T.S.H. output in the latter.

The striking summation effect in carcinogenesis of radiation followed by methylthiouracil was noted previously (Doniach, 1950) and compared with the carcinogenic summation effect of acetylaminofluorene and goitrogens first demonstrated by Bielschowsky (1944). The added interest in the present experiment is that the effective dosage of radiation from $I^{131}$ is equivalent to as little as 1100 rads X rays. This finding is of clinical interest since it suggests that X-irradiation therapy in Graves' disease carries the same risk of future carcinoma development as $I^{131}$ therapy. Also it fits the recent reports (Simpson, Hempelmann and Fuller, 1955 ; Clark, 1955) that carcinoma of the thyroid has developed in children whose thyroids were exposed to X-irradiation some years previously, in most cases the dose being well under 1000 rads. These children probably suffered a summation of direct irradiation damage to the thyroid with a secondary maintained rise in T.S.H.

In the present experiment we are comparing the effects of a dose of 190 kV. X rays delivered in 8 minutes with a dose of *beta* rays from $I^{131}$ delivered at a falling rate over a few days. One cannot therefore assume that the effective carcinogenic dose in the $I^{131}$ range was 1100 rads. One can postulate that it is likely to be well under 5000 rads, possibly 1500 to 2000 rads. In a previous experiment (Doniach, 1953), 5 $\mu$C $I^{131}$ followed by methylthiouracil for 15 months led to an incidence of adenomatous replacement of the thyroid comparable with that following 30 $\mu$C $I^{131}$. However, tumour cells were not found in extracapsular veins in any rat and malignancy was not diagnosed. The dosage range from the 5 $\mu$C $I^{131}$ was thought to lie between 380 and 2700 rads. This suggests that the effective carcinogenic radiation dose from $I^{131}$ is above 2700 rads. However, the lack of development of overt malignancy at 15 months may have been related to the number of cells exposed to the carcinogenic range of radiation. The number might well have been much smaller in the 5 $\mu$C $I^{131}$ group than in the 30 $\mu$C $I^{131}$ group. The morphological changes were strikingly suggestive of incipient malignancy and overlapped those found in the 30 $\mu$C $I^{131}$ group in which 5 examples of overt carcinoma were diagnosed in 20 animals. 30 $\mu$C $I^{131}$ depresses the mitotic response to T.S.H. of most but not all cells of the thyroid 3 to 4 months onwards after irradiation (Doniach and Logothetopoulos, 1955). After 100 $\mu$C $I^{131}$ neoplasia was considerably reduced, when presumably still fewer cells escaped post-irradiation inhibition of mitosis (Doniach, 1953).

*Further examples of carcinogenic summation of radiation with other stimuli*

Carcinomas of irradiated human skin arise on a basis of indolent ulcers which appear many years after the original irradiations (literature reviewed by Furth, 1954). Glücksmann (1951) has studied precisely the sequence of events in mouse skin exposed to about 8000 rads *beta* rays. The radiation leads to ulceration of the skin which heals and later breaks down and heals repeatedly with eventual development of malignancy in the exposed site. Malignancy was both initiated by the radiation and promoted by regeneration consequent to delayed radiation induced ischaemia. It is likely that the high dosage level of radiation was necessary not so much to initiate carcinogenesis but to induce a non-specific stimulus to tissue regeneration. Mottram (1938) obtained tumours of mouse skin by the summation of a non-carcinogenic course of benzypyrene painting (as judged by his controls) with a single exposure of 800 to 2500 rads *beta* rays. Similar results were obtained by Hamilton and Passonneau (1949) with the summation of *beta* rays and methylcholanthrene and by Boag and Glücksmann (1956) with *beta* rays and 1 : 2 : 5 : 6-dibenzanthracene or 9 : 10-dimethyl-1 : 2-benzanthracene. Lacassagne (1933) found that X-irradiation of inflammatory sites in the rabbit gave rise to connective tissue tumours. This was confirmed by Burrows, Mayneord and Roberts (1937) who obtained metastasizing sarcomas by giving 600 rads X rays to rabbits at the site of subcutaneous silica granulomas.

### CONCLUSION

It appears that maintained tissue hyperplasia, not necessarily induced by a known carcinogen, may in certain circumstances lead to neoplasia. This tendency to neoplasia is heightened by a comparatively small dose of ionizing irradiation. With regard to the thyroid the non-specifically carcinogenic stimulus to hyperplasia is an excessive secretion of T.S.H. Daily small doses of thyroid hormone partly replace endogenous thyroid hormone secretion and thereby lower T.S.H. output. This treatment applied to patients whose thyroids have been irradiated should reduce the likelihood of subsequent thyroid neoplasia.

### SUMMARY

The thyroids of 6 groups of rats, killed 15 months after the beginning of the experiment, were examined histologically for adenomas and carcinomas after the following different types of treatment: (1) Controls. (2) 30 $\mu$C $I^{131}$. (3) 1100 rads X rays to the thyroid. (4) Methylthiouracil for 15 months. (5) 30 $\mu$C $I^{131}$ followed by methylthiouracil. (6) 1100 rads X rays to the thyroid followed by methylthiouracil. A few thyroids containing small adenomas were found in the $I^{131}$ and 1100 rads X ray group. In addition, one of the X ray group showed a thyroid carcinoma. Small adenomas were present in most of the methylthiouracil group thyroids. Most of the thyroids of both the irradiated groups which were treated with subsequent methylthiouracil showed adenomatous replacement. In addition, 5 carcinomas were identified in the 24 rats given $I^{131}$ followed by methylthiouracil. Seven carcinomas were identified in the 21 rats given 1100 rads X rays followed by methylthiouracil.

Thus, a single exposure of 1100 rads X rays proved equivalent to 30 $\mu$C $I^{131}$ in its potency to initiate carcinogenesis in the thyroids of rats subsequently treated with methylthiouracil for 15 months. This finding is discussed in relation to the range of dosage to the thyroid of 2000 to 24,000 rads from 30 $\mu$C $I^{131}$ and from the point of view of the carcinogenic summation of a comparatively small dose of radiation with maintained hyperplasia induced by prolonged excessive output of pituitary T.S.H.

I am grateful to Dr. P. Howard Flanders for carrying out the X-irradiation and for criticism of the manuscript, to Dr. J. H. Logothetopoulos for help in setting up the experiment, to Mr. J. G. Griffin and colleagues for the sections, to Mr. I. T. Hinton for the photomicrographs and to Mrs. D. Gearon for secretarial assistance.

### REFERENCES

ABBATT, J. D., DONIACH, I., FLANDERS, P. H. AND LOGOTHETOPOULOS, J. H.—(1957) *Brit. J. Radiol.* **30**, 86.

AXELRAD, A. A. AND LEBLOND, C. P.—(1955) *Cancer*, **8**, 339.

BIELSCHOWSKY, F.—(1944) *Brit. J. exp. Path.*, **25**, 90.—(1955) *Brit. J. Cancer*, **9**, 80.

BOAG, J. W. AND GLÜCKSMANN, A.—(1956) 'Progress in Radiobiology'. Edited by J. S. Mitchell, B. E. Holmes and C. L. Smith. Edinburgh (Oliver and Boyd).

BURROWS, H. MAYNEORD, W. V. AND ROBERTS, J. E.—(1937) *Proc. roy. Soc.* Ser. B, **123**, 213.

CLARK, D. E.—(1955) *J. Amer. med. Ass.*, **159**, 1007.

DONIACH, I.—(1950) *Brit. J. Cancer*, **4**, 223.—(1953) *Ibid.*, **7**, 181.
*Idem* AND LOGOTHETOPOULOS, J. H.—(1955) *Ibid.*, **9**, 117.
FURTH, J.—(1954) 'Radiation Biology'. Vol. 1, Part II. Edited by A. Hollaender. New York and London (McGraw Hill Book Company ,Inc.).
GLÜCKSMANN, A.—(1951) *J. Path. Bact.*, **63**, 176.
GOLDBERG, R. C. AND CHAIKOFF, I. L.—(1950) *Endocrinology*, **46**, 91.
HAMILTON, K. AND PASSONNEAU, J.—(1949) Quoted by J. Furth (1954) above.
LACASSAGNE, A.—(1933) *C. R. Soc. Biol., Paris*, **112**, 562.
LEBLOND, C. P. AND WALKER, B. E.—(1956) *Physiol. Rev.*, **36**, 255.
MALOOF, F., DOBYNS, B. M. AND VICKERY, A. L.—(1952) *Endocrinology*, **50**, 612.
MOTTRAM, J. C.—(1938) *Amer. J. Cancer*, **32**, 76.
PURVES, H. D. AND GRIESBACH, W. E.—(1947) *Brit. J. exp. Path.*, **28**, 46.
RAPER, J. R., HENSHAW, P. S. AND SNIDER, R. S.—(1951) 'Effects of External Beta Radiation'. Edited by R. E. Zirkle. New York and London (McGraw Hill Book Company, Inc.).
SIMPSON, C. L., HEMPELMANN, L. H. AND FULLER, L. M.—(1955) *Radiology*, **64**, 840.


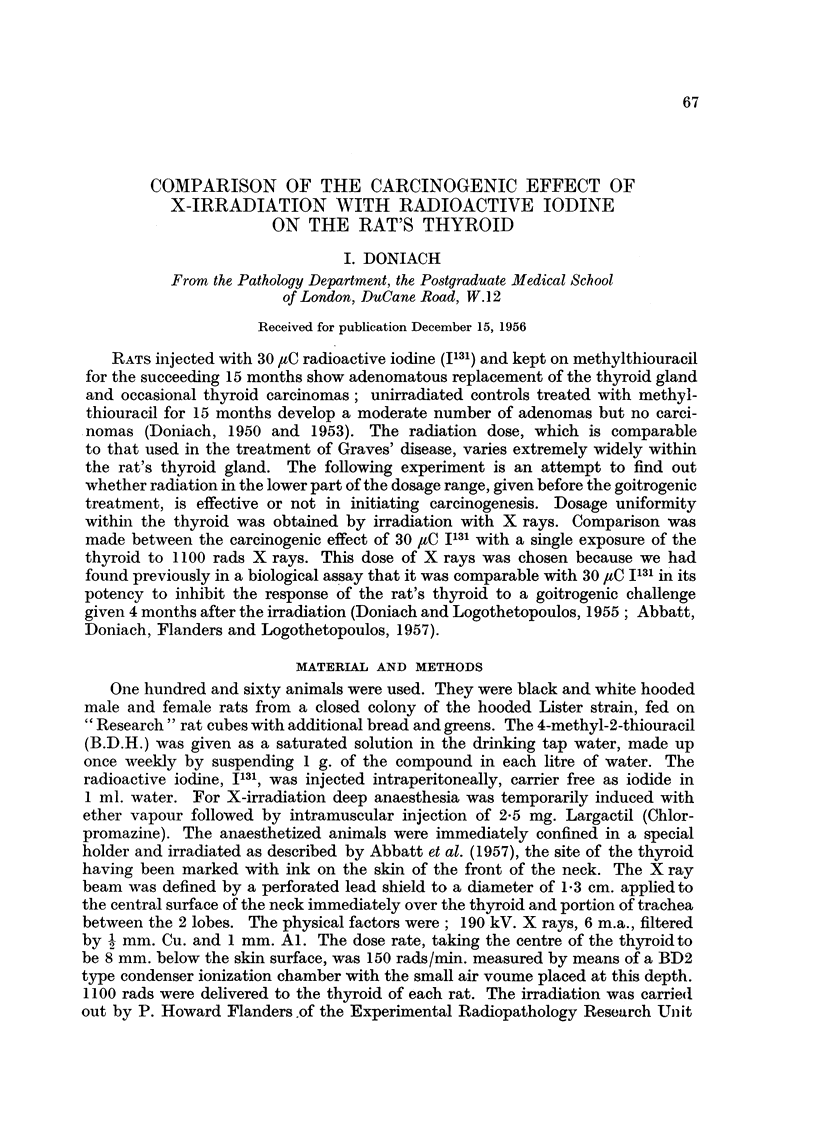

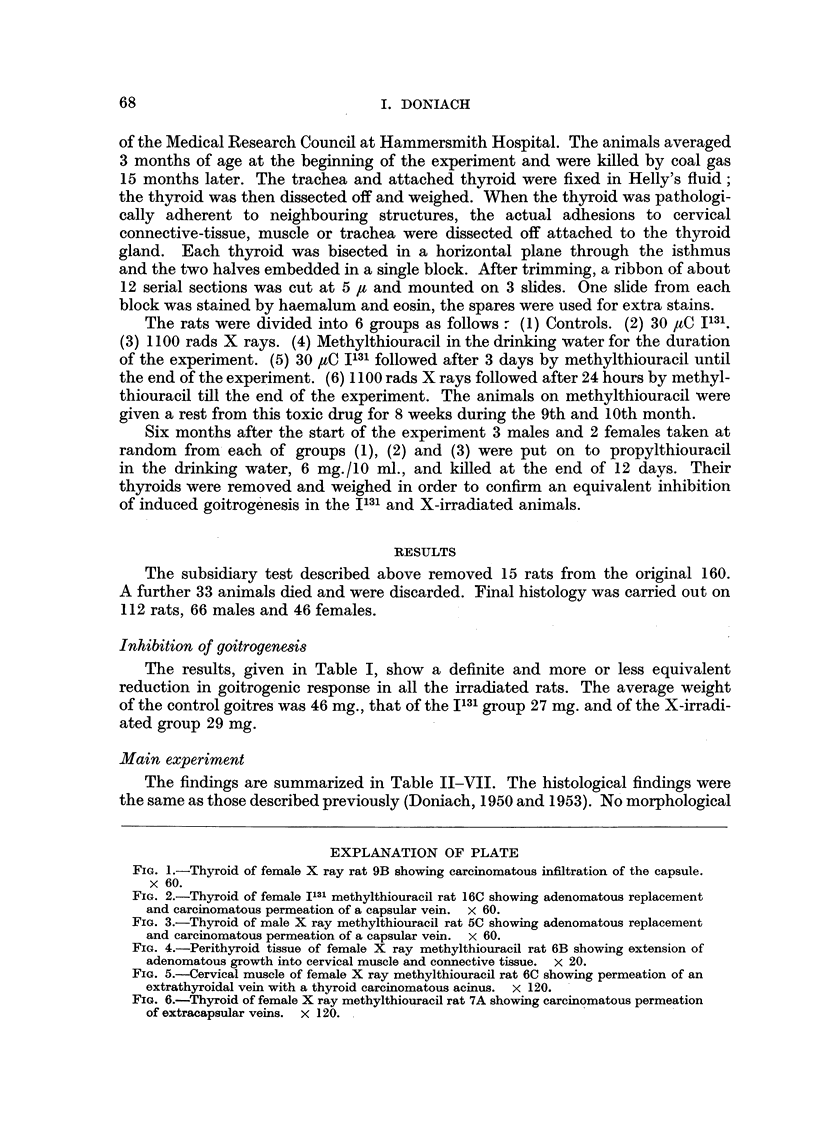

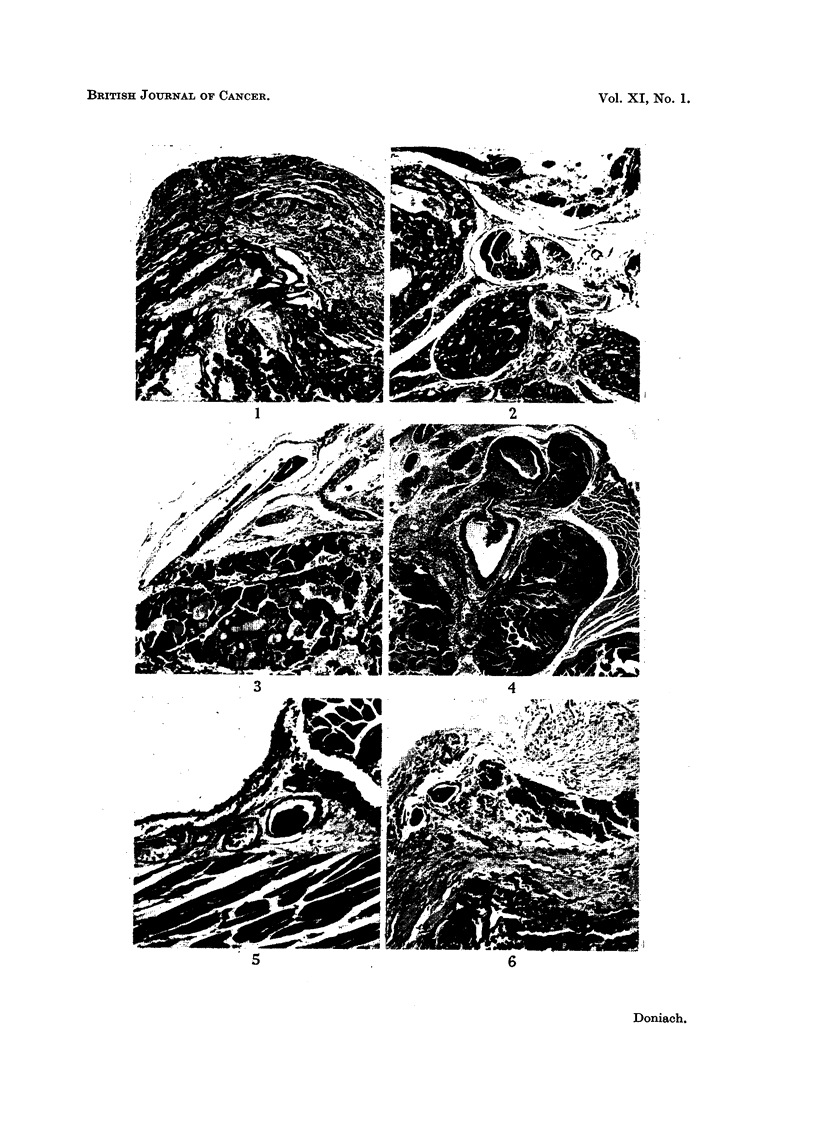

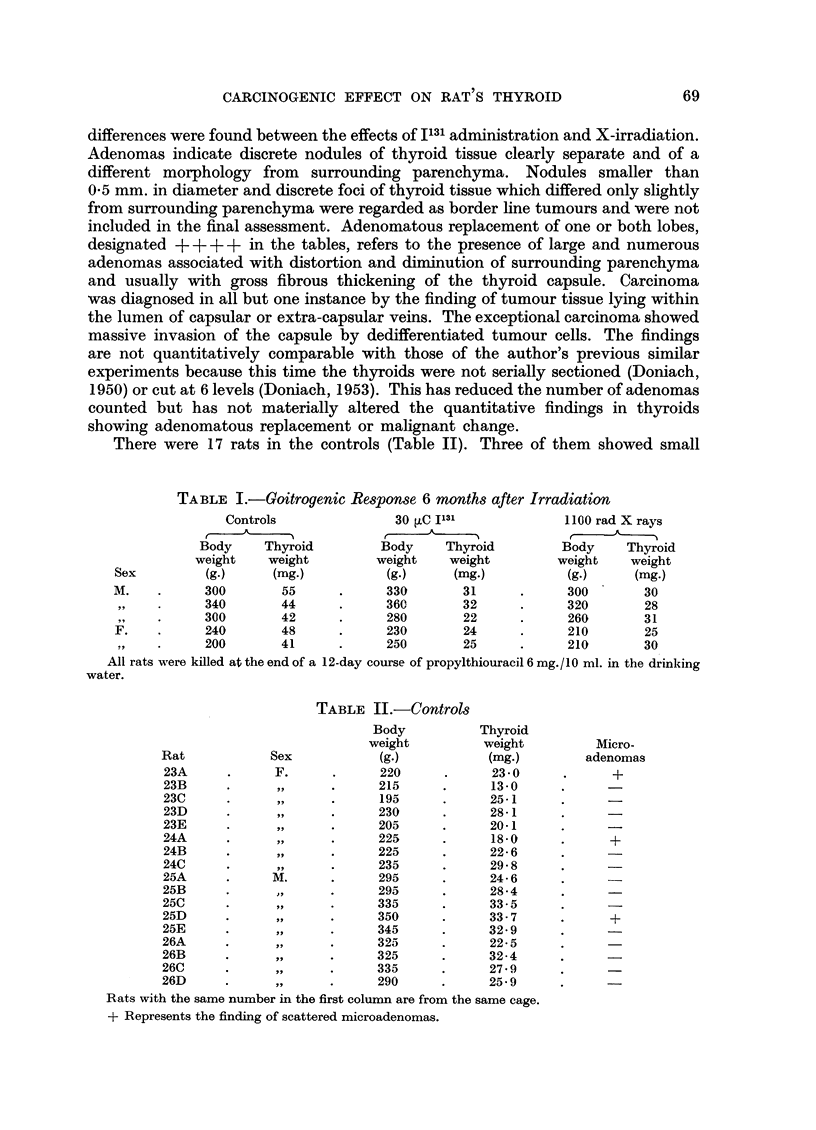

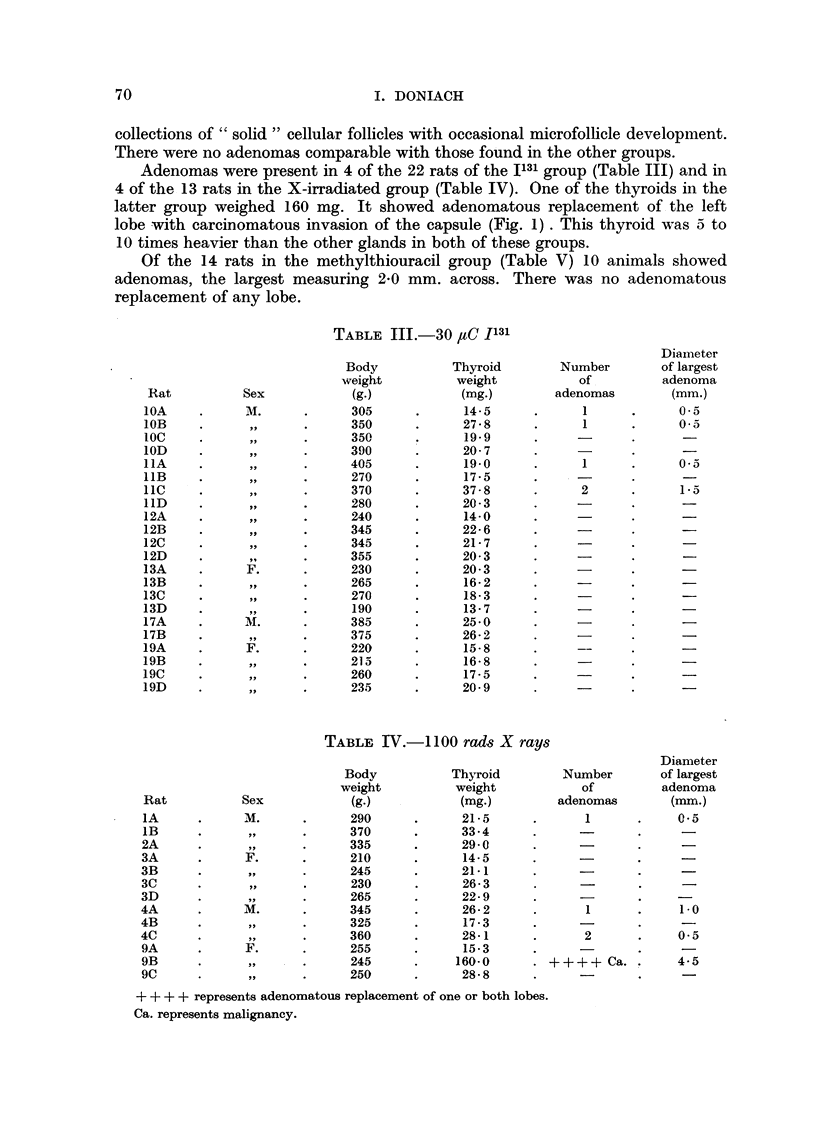

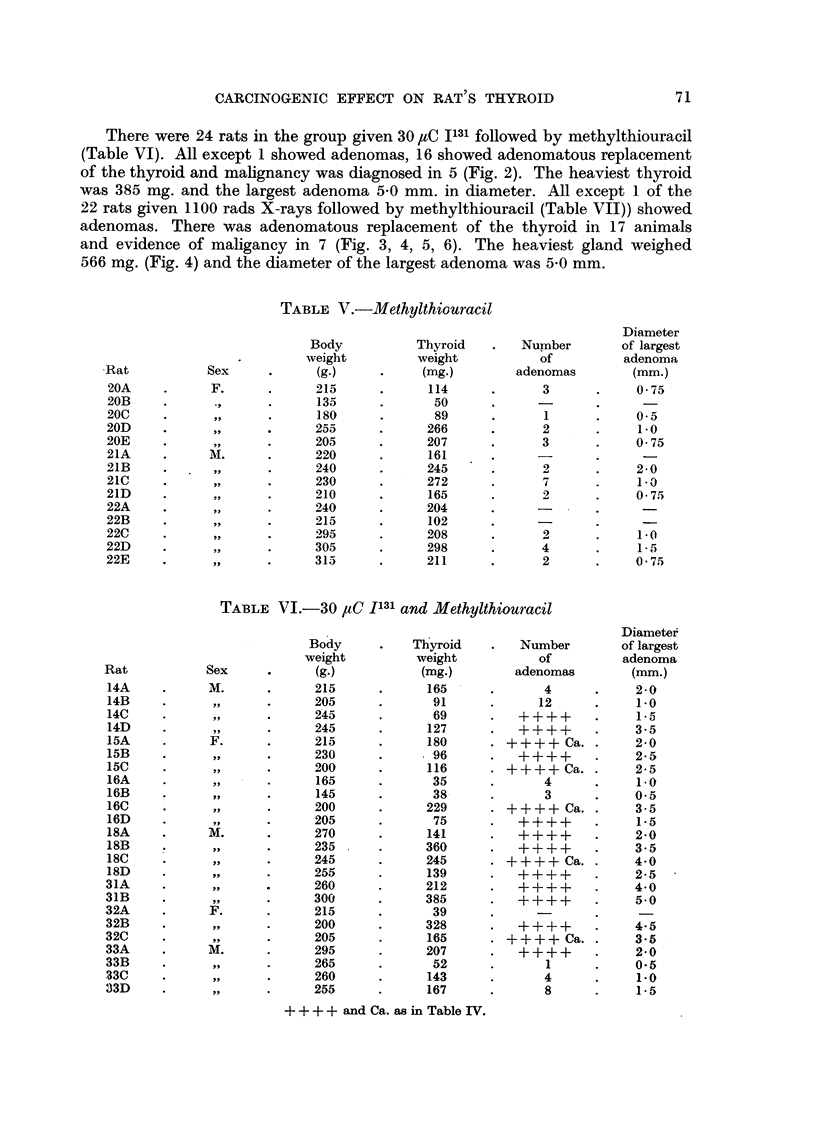

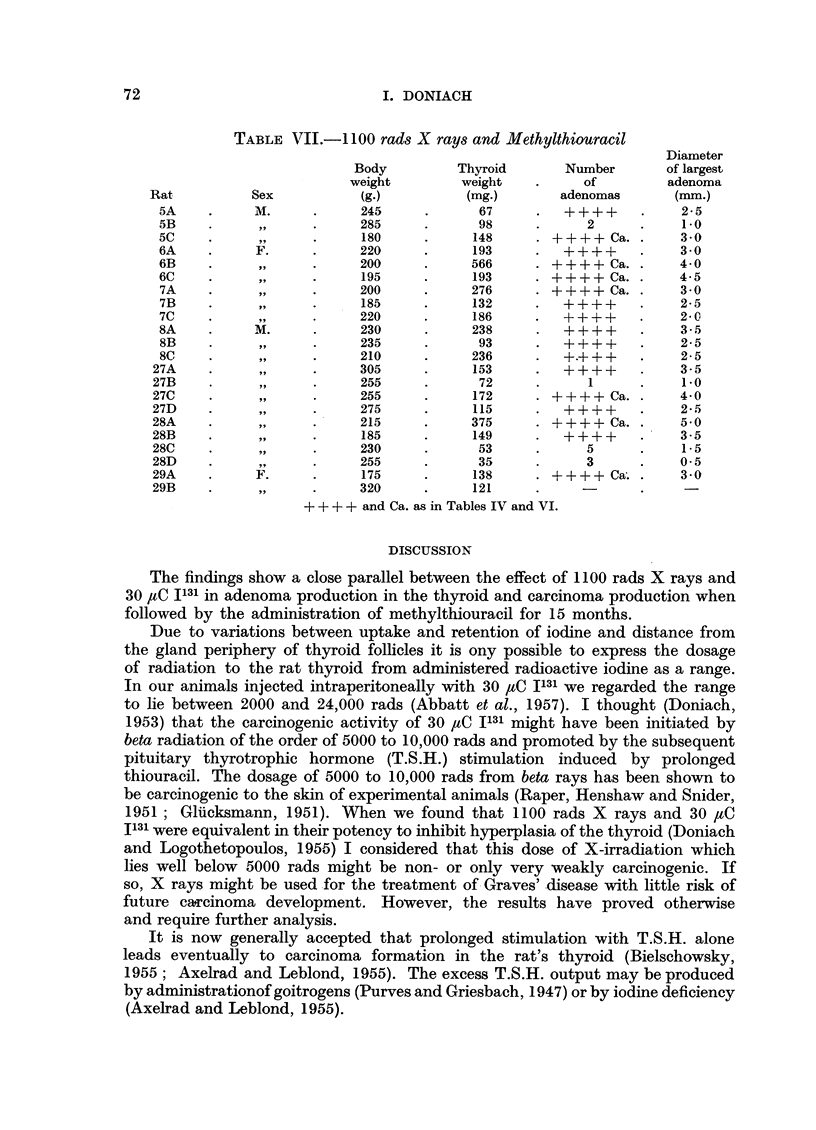

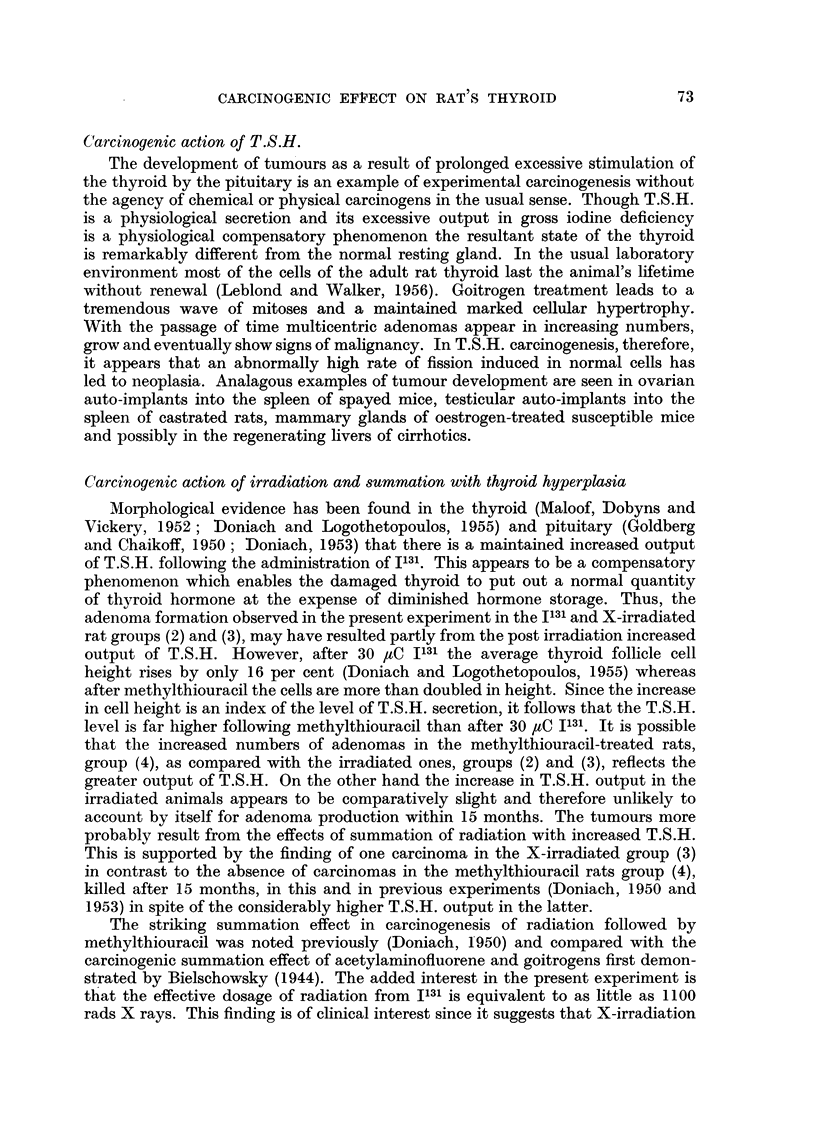

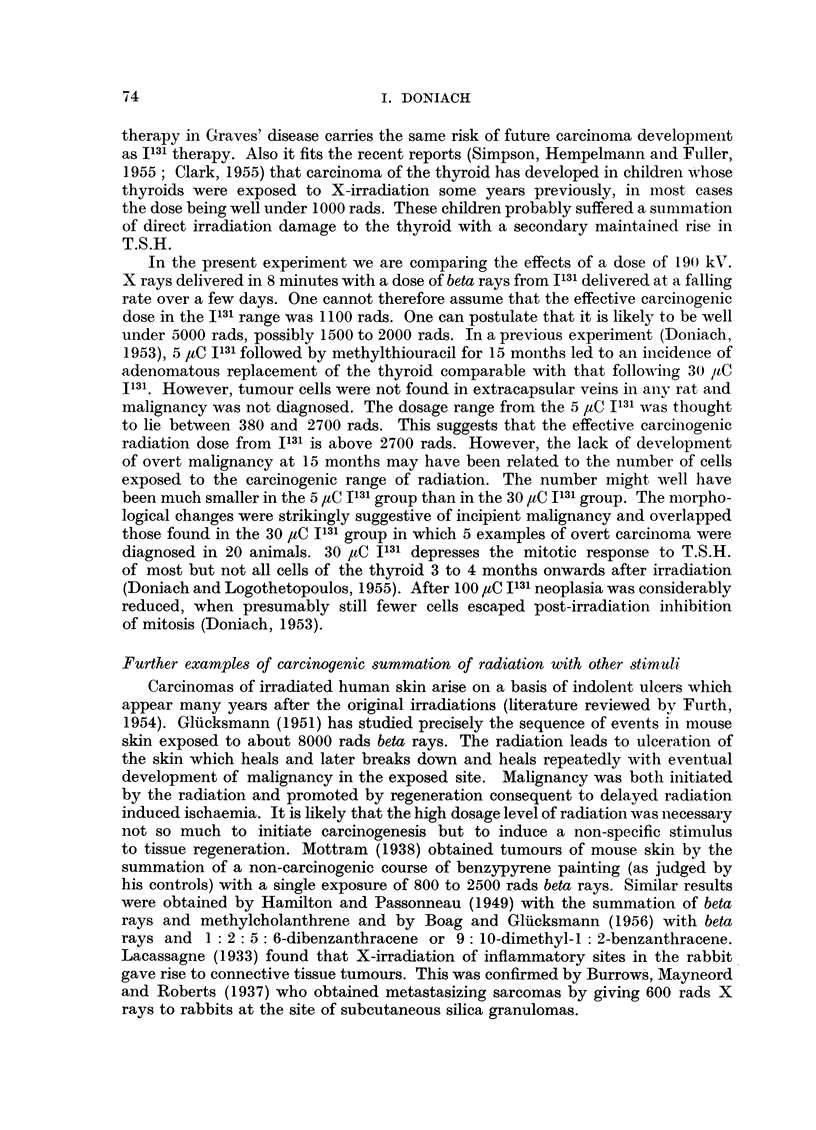

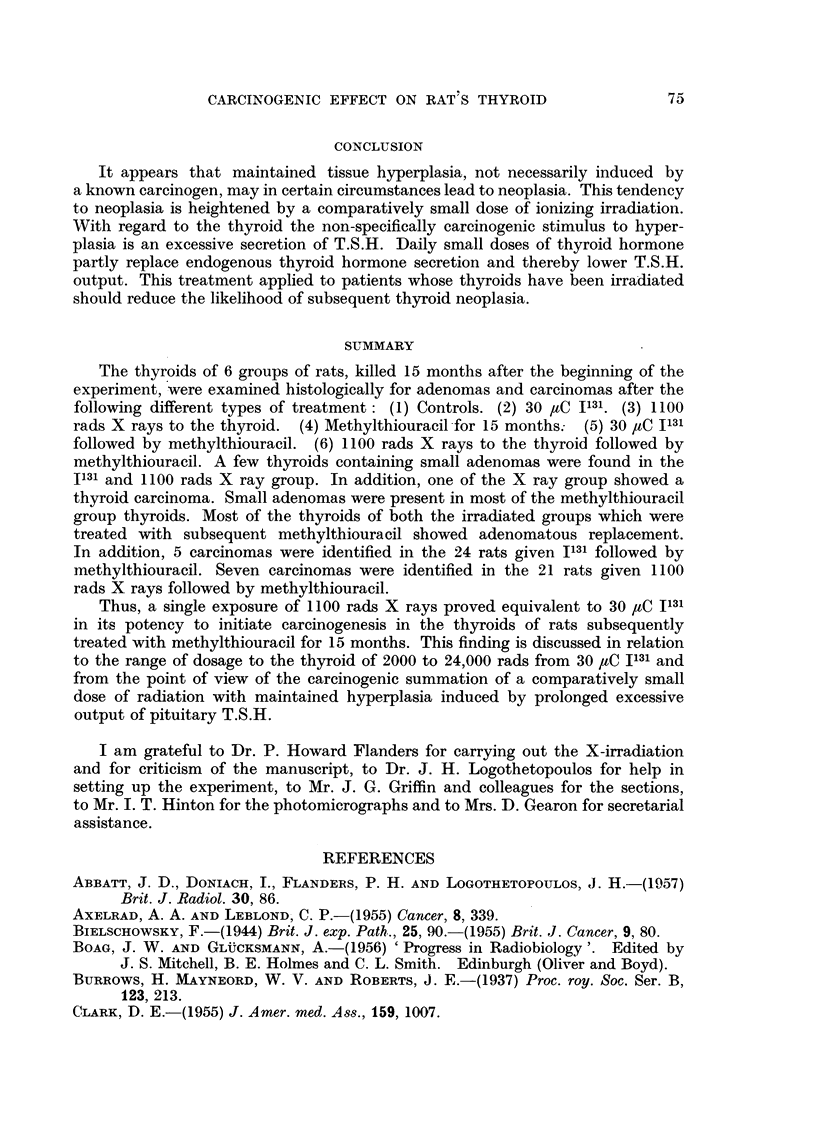

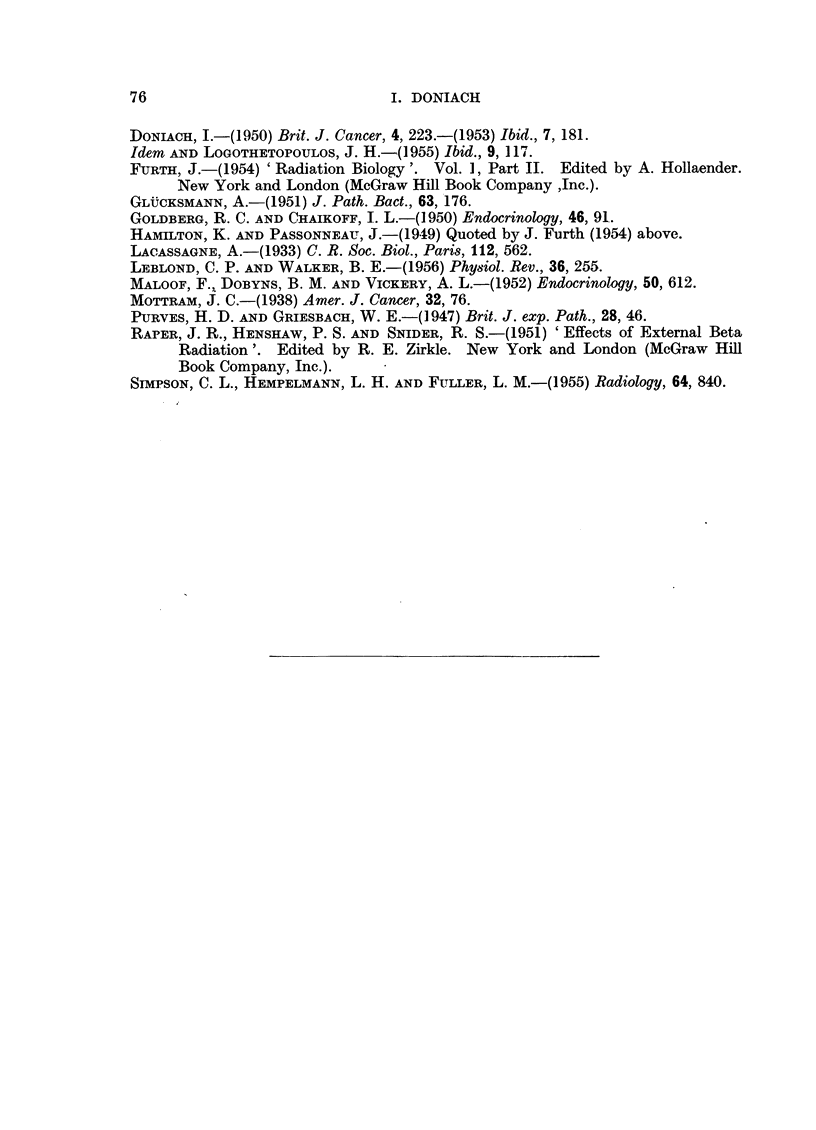


## References

[OCR_00811] AXELRAD A. A., LEBLOND C. P. (1955). Induction of thyroid tumors in rats by a low iodine diet.. Cancer.

[OCR_00825] CLARK D. E. (1955). Association of irradiation with cancer of the thyroid in children and adolescents.. J Am Med Assoc.

[OCR_00828] DONIACH I., LOGOTHETOPOULOS J. H. (1955). Effects of radioactive iodine on the rat thyroid's function, regeneration and response to goitrogens.. Br J Cancer.

[OCR_00840] LEBLOND C. P., WALKER B. E. (1956). Renewal of cell populations.. Physiol Rev.

[OCR_00842] MALOOF F., DOBYNS B. M., VICKERY A. L. (1952). The effects of various doses of radioactive iodine on the function and structure of the thyroid of the rat.. Endocrinology.

[OCR_00852] SIMPSON C. L., HEMPELMANN L. H., FULLER L. M. (1955). Neoplasia in children treated with X-rays in infancy for thymic enlargement.. Radiology.

